# Milk Powder Fortified with Potassium and Phytosterols to Decrease the Risk of Cardiovascular Events among the Adult Population in Malaysia: A Cost-Effectiveness Analysis

**DOI:** 10.3390/nu11061235

**Published:** 2019-05-30

**Authors:** Anita E. Gandola, Livia Dainelli, Diane Zimmermann, Maznah Dahlui, Patrick Detzel

**Affiliations:** 1 Nestlé Research Center, 1000 Lausanne, Switzerland; anita.Gandola@live.it (A.E.G.); diane.zimmermann@rdls.nestle.com (D.Z.); patrick.detzel@rdls.nestle.com (P.D.); 2 Università della Svizzera Italiana, 6900 Lugano, Switzerland; 3 Centre of Population Health, Faculty of Medicine, University of Malaya, Kuala Lumpur 50603, Malaysia; maznahd@ummc.edu.my; 4 Faculty of Public Health, Universitas Airlangga, Surabaya 60115, Indonesia

**Keywords:** cost-effectiveness analysis, Malaysia, potassium, phytosterols, cardiovascular, fortified food, blood pressure, cholesterol

## Abstract

This study evaluated the cost-effectiveness of the consumption of a milk powder product fortified with potassium (+1050.28 mg/day) and phytosterols (+1200 mg/day) to lower systolic blood pressure and low-density lipoprotein cholesterol, respectively, and, therefore, the risk of myocardial infarction (MI) and stroke among the 35–75-year-old population in Malaysia. A Markov model was created against a do-nothing option, from a governmental perspective, and with a time horizon of 40 years. Different data sources, encompassing clinical studies, practice guidelines, grey literature, and statistical yearbooks, were used. Sensitivity analyses were performed to evaluate the impact of uncertainty on the base case estimates. With an incremental cost-effectiveness ratio equal to international dollars (int$) 22,518.03 per quality-adjusted life-years gained, the intervention can be classified as very cost-effective. If adopted nationwide, it would help prevent at least 13,400 MIs, 30,500 strokes, and more than 10,600 and 17,100 MI- and stroke-related deaths. The discounted cost savings generated for the health care system by those who consume the fortified milk powder would amount to int$8.1 per person, corresponding to 0.7% of the total yearly health expenditure per capita. Sensitivity analyses confirmed the robustness of the results. Together with other preventive interventions, the consumption of milk powder fortified with potassium and phytosterols represents a cost-effective strategy to attenuate the rapid increase in cardiovascular burden in Malaysia.

## 1. Introduction 

In the last 50 years, due to robust economic growth and profound lifestyle changes, Malaysia has experienced a rapid nutritional transition, with increased food supply and decreasing energy expenditures linked to an obesogenic environment [1,2,3]. Although undernourishment and communicable diseases continue to affect some parts of the country, the majority of the population, urban and rural, has adopted unhealthy dietary habits, such as the excessive consumption of food high in saturated fats and salty condiments (e.g., soy sauce) [1], leading to startling increases in the prevalence of overweight, obesity, and other diet-related noncommunicable diseases (NCDs) [2,3]. In Malaysia, NCDs are a public health emergency that accounts for almost three death episodes out of four [4], and if this upward trend continues, the associated health care costs are expected to increase to “unsustainable” levels in the near future [5].

Among NCDs, cardiovascular diseases (CVDs) are the leading cause of morbidity and mortality [6] and claim one-third of all patients [7] and account for more than 459,000 disability-adjusted life years (DALYs) [8] in the country. 

Within modifiable CVD risk factors, hypertension and hypercholesterolemia play a prominent role. Hypertension increased from 14.4% in 1986 to 30.3% in 2015 [5,9] and hypercholesterolemia increased from 28.2% in 2006 to 47.7% in 2015 [5].

To maintain blood pressure (BP) at optimal levels, the World Health Organization (WHO) guidelines recommend a daily sodium intake below 2000 mg and a minimum daily potassium intake of 90 mmol or 3510 mg for the adult population [10]. The sodium-to-potassium ratio has emerged, indeed, as a better predictor of cardiovascular outcomes than sodium or potassium alone [11,12,13,14]. According to the Malaysian Adult Nutrition Surveys (MANS), the mean sodium intake has decreased from 2575 mg/day in the early 2000s [15] to 1936 mg/day in 2014 [4]. However, small cross-sectional studies applying a 24-hour urinary excretion method have revealed significantly higher estimates equal to 2860–3429 mg/day [16,17]. Unfortunately, potassium intake was not systematically collected in the MANS [18], and no studies have reported the average potassium intake of Malays/Bumiputera (62% of the population), Indians (6.3%), and/or other ethnic minorities (11%). However, a small (*n* = 254) cross-sectional study carried out in Kuala Lumpur assessing the dietary intake of Chinese adults through a 2-day 24-h dietary recall reported an average daily potassium intake of 1500 mg/day (±500) among vegetarians and 1300 mg/day (±500) among non-vegetarians [19]. Given the relevance of the Chinese ethnicity in Malaysia, this study hints at a daily potassium intake, which is likely to be far below the WHO’s recommendations for at least one person out of five [20]. Based on these results, Malaysian authorities have committed to reducing the population’s sodium intake by 30% by 2025 [21] and have recognized the essential role of potassium and the potential benefits of food fortified with this micronutrient [22]. 

Although the WHO has not recommended any daily intake of phytosterols (i.e., plant sterols and stanols, hereafter phytosterols) for the general population thus far, these naturally occurring compounds contribute to the reduction of low-density lipoprotein cholesterol (LDL-c) in plasma concentration [23,24,25], which lowers ischemic heart disease risk [26]. Neither MANS [18] nor other research has quantified phytosterols intake in the Malaysian population.

This study evaluates the potential health and economic effects of the consumption of a milk powder product fortified with potassium and phytosterols to lower systolic blood pressure (SBP) and LDL-c, respectively, and the ensuing risk of CVD events. 

## 2. Materials and Methods

A cost-effectiveness analysis from a governmental perspective was conducted to evaluate the potential cost savings and the number of prevented myocardial infarction (MI) and stroke if a milk powder product fortified with potassium (+1050.28mg/day) and phytosterols (+1200 mg/day) was consumed every day by the 35–75-year-old population in Malaysia. Among modifiable CVD risk factors, dietary patterns play a major role. Additional potassium (K) intake may contribute to lower SBP, and additional phytosterols intake may contribute to lower hypercholesterolemia (LDL-c); both decrease the relative risk (RR) of cardiovascular events such as MI and stroke.

**Figure 1:**
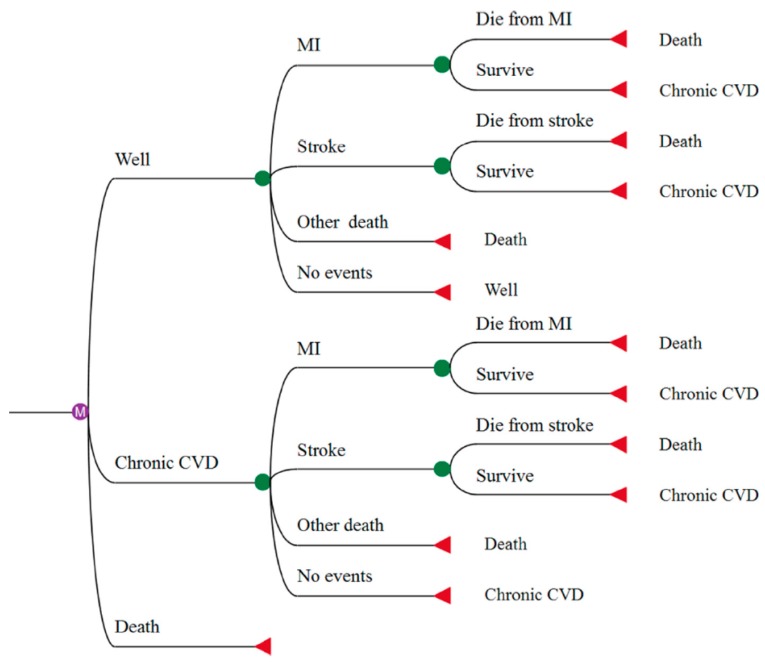
Decision tree representation of one branch of the Markov model structure for each arm. The model was based on the structure of Li [27]. The “well” and “chronic cardiovascular disease (CVD)” states are populated by individuals without and with prior myocardial infarction (MI) or stroke, respectively. The initial distribution of the population was set as follows: 3.83% of the individuals started in the “chronic CVD” state, and the remaining 96.17% started from the “well” state [28]. Individuals in the “well” state can remain in their state or progress to “chronic CVD” in the case of a first-ever MI or stroke. In each cycle, individuals in the “well” and the “chronic CVD” states may experience an MI or stroke and are characterized by the same survival probabilities as other individuals in their state, regardless of their medical history. “Other death” accounts for all individuals who die of causes that differ from CVDs.

A Markov model, based on the structure of Li et al [27], was created in TreeAge Pro 2018 to model the effects within a time horizon of 40 years. The comparator used was a do-nothing option. Both strategies (milk fortified vs do-nothing option) were modeled with the same branch structure but featured different transition probabilities (Figure 1). The initial distribution of the model population was set as follows: 3.83% of the individuals started in a “chronic CVD” state, and the remaining 96.17% started from the “well” state [28]. Uncertainty and assumptions due to the notable lack of country-specific epidemiologic data reflecting the ethnical heterogeneity of Malaysia were addressed through deterministic and probabilistic sensitivity analyses. Due to our reliance on secondary data, no institutional review board or ethics committee approval was required for this study. 

### 2.1. Stroke and MI Incidence per Annum and Mortality Rates

To populate the different branches of the model, we relied on different sources, encompassing local epidemiologic and clinical studies [6,29,30,31,32,33,34,35,36,37] and grey literature (e.g., annual reports of the Malaysian ACS and Stroke Registry [38,39,40,41,42,43], clinical practice guidelines [44,45,46,47,48,49], and statistical yearbooks [20,50]). 

More specifically, the 30-day mortality rate due to an MI for the well population and for the chronic CVD population was estimated by stratifying the survival/death probabilities according to the MI typology (ST-elevation myocardial infarction, non-ST-elevation myocardial infarction and unstable angina) weighted by their respective frequencies [41,42] and the age cohort of the individuals affected [30]. Similarly, to estimate the 28-day mortality rate due to stroke for both the well and the chronic CVD population we relied on an average of the probabilities stratified according to stroke typology (ischemic, intracerebral hemorrhagic, and subarachnoid hemorrhagic) weighted by their relative frequency [33]. To differentiate the mortality rates according to age, we adopted the approach used in Dainelli et al. [51]: annual increases in mortality rates were computed by subtracting the 28-day to the 1-year survival probability estimates and then averaged according to their relative frequencies. The same increase was adopted for 5-year age cohorts by applying the following formula:

$$P_{t}=P_{0}\left(1+annual\;rate\right)t$$

where *P*_0_ is the baseline probability and *t* indicates the difference in years from the occurrence of the first-ever stroke. To model the compounded effect of an increase in potassium and phytosterols intake, we assumed a linear additive effect of the two food compounds in reducing the RR of CVD events for the individuals consuming the fortified milk powder. Although a decrease in blood cholesterol would also be expected to contribute to a slight reduction in BP [52], we assumed any interaction between the two risk factors to be minimal and used a conservative approach by considering each effect separately. Incidence and mortality data used in the do-nothing branch of the model are reported in Table 1. The RR reduction for stroke and MI generated by a 1050.28 mg/day increase in potassium intake, a 1200 mg/day increase in phytosterol intake, and their compounded (additive) effect is presented in Table 2.

### 2.2. Potassium Effect on SBP and SBP Effect on CVD Risk

The beneficial effect of potassium intake in lowering SBP is unanimously recognized by the literature [11,12,13,14,53,54,55,56,57,58]. In the absence of clinical trials testing the effectiveness of increased potassium intake on SBP in Malaysia, we relied on an intervention study carried out in China [59], in which a heterogeneous adult population (normotensive, pre-hypertensive, and hypertensive) consumed 60 mmol of potassium chloride tablets. Compliance among subjects was measured assuming 80% recovery and through 24-h urine samples repeated in each individual, which is widely considered the only method guaranteeing an unbiased estimate and a high degree of confidence in measuring potassium intake [60,61,62,63]; results indicated a dietary intake of approximately 26 mmol/day, corresponding to approximately 1 g of potassium. The subsequent variation in SBP amounted to −6.38 mmHg after 6 weeks and −3.68 mmHg after 12 weeks, with no statistically meaningful difference when stratifying for gender; to adopt a conservative approach, the latter value was used to quantify the impact of an increase of 1 g in potassium intake on SBP in line with the meta-analysis of Aburto et al. [64], who report an estimate of −3.65 mmHg. 

The results of the aforementioned intervention study [59] were extended to the whole population of Malaysia on the hypothesis that even if characterized by a significant variation in dietary patterns due to socioeconomic and cultural (e.g., religion) causes, there is no evident reason to assume different effectiveness of potassium intake on SBP across ethnic groups. We expected that increasing potassium intake would contribute to improving the sodium-to-potassium ratio. An optimal effect would be achieved by decreasing sodium and increasing potassium simultaneously. However, the sodium level in this paper was considered fixed.

Finally, the medical literature unanimously recognizes the proportional relationship linking SBP and CVD risks [55,65]. In the absence of local data on the effect of a reduction in SBP on CVD incidence and mortality, we relied on the Asia Pacific Cohort Study Collaboration [66] due to its geographical affinity, the inclusion of both relevant endpoints (i.e., stroke and MI), and the availability of age-specific estimates [67] to model the aforementioned relationship in our study. Hyperkalemia (i.e., excessive potassium level in blood serum) and related disease risks have been excluded from the model due to the rareness of the disease in the general population [68].

### 2.3. Phytosterols Effect on LDL-c and LDL-c Effect on CVD Risk

The scientific evidence supporting an inverse relationship between phytosterols and LDL-c is robust and consistent [23,24,25,69,70,71,72,73,74,75,76,77], and the relevance of an elevated LDL-c concentration in the bloodstream has been confirmed as a risk factor for CVDs [65]. The dose-response of an increase in phytosterol intake of 1200 mg/day, which corresponds to the amount considered for this study, on LDL-c levels varies between −4.8% and −10.5% [24,25,73]. Due to the absence of local studies, we relied on the lower value, namely, 4.8%, as reported by Yang et al. [24], to be conservative. 

A nonlinear relationship linking phytosterols and LDL-c has been robustly identified in the clinical literature [25,78,79]; more specifically, once the threshold of a 2.5 g intake has been reached, a plateau effect is generated, for which marginal increments of phytosterols intake are associated with nonsignificant decreases in LDL-c (ibid.). Given that the additional daily phytosterols intake whose health and economic effects are being enquired about in this paper amounts to 1.2 g and that we expect this variation to exert an impact on LDL-c, we are implicitly assuming the average phytosterols intake characterizing the modeled population to be lower than 1.3 g/day, because any baseline intake higher than 1.3 g/day would make a 1.2 g/day increase non-significantly related to LDL-c decrements (1.3 + 1.2 = 2.5 g/day).

As the findings presented in the most recent National Health and Morbidity Survey hint at an overall prevalence of hypercholesterolemia amounting to approximately half of the population (47.7%), reaching the level of 68.8% in adults 55–59 years of age and not differing substantially across the 3 most populous ethnic groups of the country [4], this hypothesis appears warranted. 

As for the effectiveness of potassium on SBP, we assumed no ethnic difference in the effect of variations in phytosterols intake on LDL-c levels, given that no evidence of the contrary has been provided by the literature, according to our review. Again, given the unavailability of local data, we relied on the study by Yang et al. [24] to model the effect of an increased phytosterols intake on LDL-c levels and CVD incidence. As for the effect of a lower SBP on CVD risk, RR reductions were calculated for different age cohorts. Data on the compound effectiveness in reducing the occurrence of CVD events are summarized in Table 2.

### 2.4. Cost and Utilities 

All costs were inflated to 2016 levels by estimating the cumulative inflation rate relative to consumer prices [80] and converted to international dollars (int$) at the 2016 exchange rate int$1 = 1.425 Malaysian ringgit (MYR) [81]. The price attributed to a portion of milk powder was equal to int$0.5. Given the governmental perspective adopted, no productivity losses caused by the cardiovascular events were included. 

Due to the important heterogeneity characterizing health preferences, in cultural values and the perception of health and illness, use of population-specific quality-adjusted life year (QALY) estimates whenever available [82,83,84] is crucial. Thus, we relied on local studies to derive QALY estimates for the health states modeled [85,86,87,88]. Future streams of costs and utilities were discounted at a 3% rate [89].

All costs [7,82,90,91,92,93,94,95,96,97,98,99,100,101] and utilities [85,86,87,88] related to a cardiovascular event and to the treatment of hypertension and hypercholesterolemia are reported in Table 3. 

## 3. Results

### 3.1. Base Case Results

With an incremental cost-effectiveness ratio (ICER) equal to int$22518.03/QALY gained, the intervention can be classified as very cost-effective according to the WHO conventional threshold, which quantifies the country’s willingness to pay (WTP) for a QALY with its gross domestic product (GDP) per capita (very cost-effective if ICER < GDP per capita; cost-effective if ICER equals to 1–3 times GDP per capita; not cost-effective if ICER > 3times GDP per capita) [88]. Given a population of approximately 11.1 million in the age group of interest (35–75 years) [20] and based on the MI and stroke incidence reported in Table 1 and on the RR reduction reported in Table 2, according to our findings, if adopted nationwide, the milk powder fortified with potassium and phytosterols would help prevent at least 13,400 MI (−7%), 30,500 strokes (−20%), and more than 10,600 and 17,100 MI- and stroke-related deaths over 40 years, respectively. The discounted cost savings generated for the health care system by those who consume the fortified milk powder would amount to approximately int$8.1 a year per person, which correspond to the 0.7% of the total yearly health expenditure per capita, amounting to int$1040 in 2014 [102]. 

### 3.2. Sensitivity Analyses

**Figure 2:**
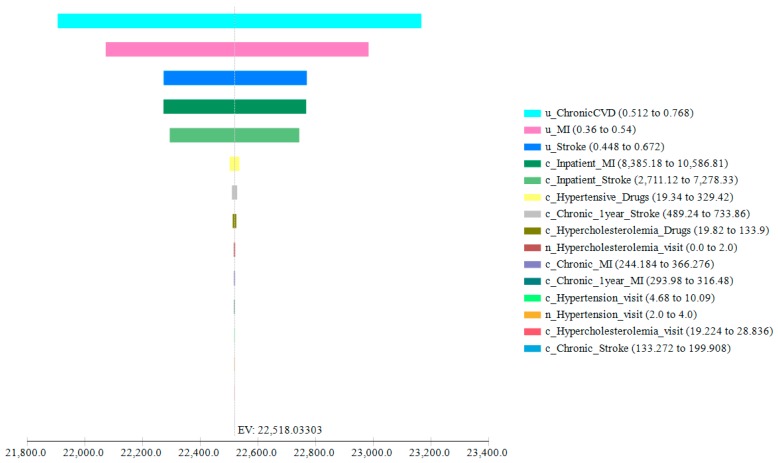
Tornado diagram. Bars represent the relative importance of the input parameters on the expected value (EV): the larger the bar, the higher the impact of that item. In the legend, the inputs to whom the bars correspond are in order of importance. The initial letter indicates the type of input: “u” stands for utility, “c” for cost, and “n” for number.

We performed deterministic and probabilistic sensitivity analyses to evaluate the impact of uncertainty on the base case estimates. In the deterministic sensitivity analysis (tornado diagram, Figure 2), we applied the appropriate ranges where available (Table 3) and ±20% variations to all the other inputs. The utilities of suffering from chronic CVD, of having a stroke or an MI, and the cost of being hospitalized after those events had the largest impact on the final outcome. 

**Figure 3:**
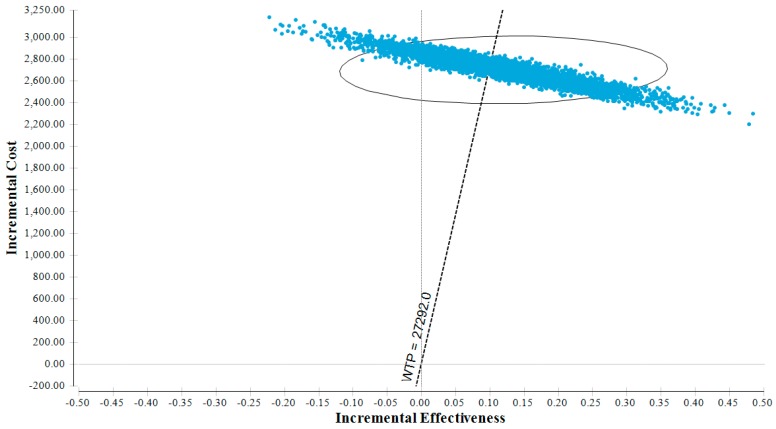
Monte Carlo simulation. Dots represent the values of the incremental cost-effectiveness ratio in 5000 simulations carried out to consider the uncertainty input parameters. The ellipse represents 95% of all data points. The line passing through the intersection of the axes represents the willingness to pay (WTP), corresponding to the gross domestic product per capita; 58.3% of the dots are located in the northeast quadrant on the right of the WTP line, that is, due to stochastic and parametric uncertainty, we expect the fortified milk powder to be a cost-effective intervention in 58.3% of the cases.

In the probabilistic sensitivity analysis (Monte Carlo simulation, Figure 3), all costs and resources used were modeled using a gamma distribution, and a beta distribution was applied to utilities [103]. Mortality estimates by age group were modeled with a normal distribution where the mean corresponded to the base estimates and a 5% standard deviation was applied. According to the 5000 simulations carried out to consider the uncertainty input parameters, the fortified milk powder was a cost-effective intervention in 58.3% of the cases.

## 4. Discussion

### 4.1. Principal Findings

Malaysia is still characterized by “low awareness, low treatment and poor control of hypertension” [104]. Notwithstanding the highly subsidized public health system granting mass screening to every individual [97], a high proportion of Malaysians is characterized by undiagnosed NCD risk factors; for instance, “for every two diagnosed hypertension, there are three undiagnosed hypertension, a ratio of 2:3” [4]. According to our review of the literature, this study is the first to show what would be the health and economic impacts of increasing potassium and phytosterols intake through consumption of a fortified milk powder product to reduce BP and LDL-cholesterol levels to prevent cardiovascular diseases in the adult population of Malaysia. 

### 4.2. Public Health Implications

The consumption of milk and dairy foods (except for butter) has been shown to decrease several CVD risk factors such as BP or arterial stiffness [105], and to lower the risk of CVD, especially stroke [106]. Notably, a recent study conducted in Singapore demonstrated that daily milk drinkers had a significantly lower risk of hypertension [107]. For 28% of the 35–75-year-olds who regularly consume milk [108], the switch to this fortified milk powder would, therefore, contribute to delivering a beneficial effect without increasing the total caloric intake. 

In addition to health benefits, the consumption of extra doses of potassium and phytosterols would produce significant economic benefits. Even if Malaysia has an inclusive public healthcare service with universal health coverage, 70–80% of the population relies on private facilities [109,110]. In this study, we assumed the same cost of care at private hospitals/clinics and at tertiary public hospitals [111]. In reality, however, private charges are usually three to four times higher than public costs. Thus, if the present analysis had been conducted from a patient perspective, significant reductions in out-of-pocket costs (and more important, costs generated in the private healthcare sector) due to a decreased incidence in CVD events would have substantially increased the economic benefits generated by the intervention. 

Given the governmental perspective adopted, the cost of the milk powder product would not have been required as an input because the purchasing decision and related expense is performed by consumers. However, its exclusion would have generated a scenario in which the benefits of the intervention were realized at no cost, nullifying the validity and the interest in conducting a cost-effectiveness analysis. Therefore, a symbolic price equal to int$0.5/day was attributed to the milk powder. This amount can be interpreted as a potential subsidy that would be cost-effective for the Malaysian government to reimburse to citizens to alleviate the cost of their purchase, or, alternatively, the price of a public campaign aimed at encouraging individuals to introduce more potassium and phytosterols in their diets. A real market price could be higher than int$0.5 per portion without impacting the validity of the proposed conclusions because consumers’ WTP and purchase decisions do not depend on the health benefits generated by a product exclusively but also on the sensory rewards (i.e., “the hedonistic rewards” [112]) generated by the consumption of such products [113,114]. The presence of such an additional component clearly distinguishes nonfortified and fortified food from pharmaceuticals and dietary supplements (generally in the form of pills and tablets) and exerts a critical impact on the formulation of fortified food (e.g., the bitterness of potassium) and on the WTP for the nutritional technology considered. Although the discipline of nutrition economics has acknowledged the severe dangers associated with excluding the impact of nutritional interventions on taste when assessing their health-related impact [114], no consensus has emerged thus far on the appropriate methodology through which the hedonistic component (in its role of limiting the extent of the fortification and in terms of its interaction with the WTP for health outcomes) should be included and quantified when assessing the cost-effectiveness of a nutritional intervention. Given that such exploration was beyond the scope of this paper, we limited our conclusions to acknowledging the proportion of the full WTP for nutritional technology that would be very cost-effective (as per the WHO definitions) to finance due to its beneficial outcomes on cardiovascular diseases.

### 4.3. Comparison with the Literature

According to our review of the literature, no study has estimated what would be the health and economic effects of nutrition interventions aimed at improving cardiovascular health in Malaysia. Findings from other countries have provided overall positive results, suggesting that nutrition interventions, together with other strategies, such as early diagnosis and national hypertension treatment programs could be valid allies in fighting NCDs [115,116]. For example, two recent studies from China, a country geographically proximal to Malaysia, one study evaluating an educational program to lower salt intake of schoolchildren’s families in an urban area [27], and one study modeling the long-term cost-effectiveness of milk powder fortified with potassium for the 50–79-year-olds who regularly consume milk [51], proved the cost-effectiveness of the interventions. Different from those studies, which targeted only SBP as a risk factor (either through a reduction of the salt intake [27] or an increment in the consumption of potassium [51]), this study evaluated the effect of potassium and phytosterols together to focus on SBP and cholesterol. 

Similarly, two European studies have provided evidence on the cost-effectiveness of margarine enriched with plant sterols to prevent CVD [24,117]. The first study was carried out in Germany from a health-insurer perspective on a representative sample of the 30–79-year-old population and featuring a decrease in coronary heart disease (CHD) risk and estimated a reduction of 117,000 CHD cases and a cost reduction of €1.3 billion over 10 years [117]. The second study adopted the perspective of the British National Health Service (NHS) and focused on 45–85-year-olds with hypercholesterolemia [24]. The second study found that the daily consumption of enriched spread was cost-effective in reducing CVD risks for men and older age groups (and less cost-effective for younger cohorts) in a scenario in which NHS paid the excess cost of the enriched spread. If the consumers bore the full cost of enriched spreads, as in our study, the intervention would be cost-saving. 

Finally, and different from the aforementioned findings, a study carried out in the Netherlands evaluated the cost-effectiveness of functional foods containing phytosterols/phytostanols—in addition to statins—to prevent cardiovascular diseases among the population of 35–75-year-olds eligible for use and found this strategy to be not cost-effective [118].

### 4.4. Strengths and Limitations

The most substantial strength of this study is the adoption of a conservative approach. This model simulated the effect of an additional daily potassium and phytosterols intake without considering the potential cumulative effect on the reduction of risk-factor levels generated by repeated intakes. For example, 1050.28 mg/day of potassium generate an effectiveness of −3.68 mmHg on SBP after 12 weeks [59] but given that no robust evidence has thus far been collected on the capacity of potassium to generate successive decreases in SBP, we assumed the aforementioned dose-response to be the effectiveness of a whole year of repeated intake. The same approach was adopted for phytosterols. Moreover, we focused on only the effectiveness of potassium on SBP, excluding other beneficial effects caused by an increased potassium intake such as lower urinary calcium excretion, decreased risk of kidney stones, support to the function of highly irrigated organs (e.g., heart, kidney, and nervous system), and prevention of bone demineralization [119,120,121,122]. Additionally, in general, milk and dairy products tend to beneficially contribute to skeletal health [123,124] and therefore appear to be ideal carriers for potassium. In addition, as shown by the literature [24], nutritional interventions produce incommensurably higher benefits for specific subgroups; therefore, we expect the fortified milk powder to be even more cost-effective for individuals at risk of CVDs and for older age groups. Another feature contributing to the conservativeness of this model is the exclusion of indirect costs, which can be substantial when considering CVDs (e.g., productivity costs) [125] but were not considered in this study due to the governmental perspective adopted. Finally, we must emphasize that, by contrast with discrete time simulation models [126], where the relevant risk factors evolve over time, we assumed the prevalence of hypertension and hypercholesterolemia and the estimates of MI and stroke mortality and morbidity remain constant over the time horizon of the model for a fixed age cohort. The dynamic feature of the model is instead provided by the increase in the age of the population and the corresponding increases in the probabilities of mortality and morbidity for the modeled cardiovascular events. Given the long-term trend in NCDs characterizing the country [5], this perspective is conservative but nonetheless necessary because of the scarcity of available data. 

The first limitation of this study was the use of multiple sources of data for the input parameter values of the model. For example, we relied on a UK study for the effectiveness of phytosterols [24], on a Chinese study to model the effectiveness of potassium [59], and on an Asian study to model the effect of a decreased SBP on CVD risks [66]. Another methodological limit of the model concerns the lack of ethnic differentiation across individuals, which would have improved the accuracy of the model given the ethnic heterogeneity characterizing the country. However, this differentiation would have required stratified data on CVD incidence, mortality, and costs, which were—unfortunately—unavailable. In any case, the cross-ethnic difference in the prevalence of the two risk factors included in our analysis was not statistically significant according to Malaysian public health authorities [4].

This study has limitations from a modeling perspective as well. The first limitation is our use of a do-nothing option. More ideal would have been to compare the fortified milk powder with a relevant comparator, such as nonfortified milk powder/standard milk; however, the results would have been less applicable to the whole population because of the necessity to restrict the analysis to the subgroup of milk drinkers in Malaysia. Second, given that phytosterols exert their beneficial impact on LDL-c in 2 to 3 weeks [127] and that the clinical trial used to measure the impact of potassium on SBP referred to a time horizon of 12 weeks [59], the choice of a Markov model featuring 1-year cycles is coherent. Still, the epidemiologic literature has traditionally argued that a certain lag time is necessary to witness the effect of a decrease in risk factors on the occurrence of CVD events, although no consensus has been reached on its size, which varies from 1–5 years [67,125,128,129]. Third and last, the choice of referring to the Malaysian GDP per capita as an estimate of its WTP for a QALY was coherent with the WHO guidelines [89] but contrasted with a local study revealing much lower estimates [130].

## 5. Conclusions

Together with other preventive interventions, the consumption of milk powder fortified with potassium and phytosterols represents a cost-effective strategy for the Malaysian government to attenuate the rapid increase in cardiovascular burden among the adult population. 

## Figures and Tables

**Table 1:** Stroke and MI incidence per annum, mortality rates.

	**Age Group**	**Stroke Incidence** [32,35,38,45]	**28-day Mortality Risk of Stroke (%)** [33,43]	**MI Incidence** [39,42]	**30-day Mortality Risk of MI (%)** [30,42]	**Non-CVD Mortality [20,50]**	
**Well**	35–39	425/100,000	17.80	11.4/100,000	7.10	74.6/100,000	
	40–44	850/100,000	18.52	43.7/100,000	12.55	74.6/100,000	
	45–49	850/100,000	19.27	43.7/100,000	12.55	2365.2/100,000	
	50–54	1700/100,000	20.05	101.2/100,000	12.55	2365.2/100,000	
	55–59	1700/100,000	20.86	101.2/100,000	12.55	4655.8/100,000	
	60–64	2000/100,000	21.70	141.8/100,000	31.35	4655.8/100,000	
	65–69	3000/100,000	22.55	141.8/100,000	31.35	6946.4/100,000	
	70–74	3500/100,000	23.42	173.2/100,000	31.35	6946.4/100,000	
	75–79	7800/100,000	24.33	173.2/100,000	31.35	6946.4/100,000	
**Chronic**	**Age group**	**Stroke incidence**	**28-day mortality risk of stroke (%)**	**MI incidence**	**30-day mortality risk of MI (%)**	**Stroke: non-CVD mortality**	**MI: non-CVD mortality**
	35–39	13,000/100,000	21.43	24,400/100,000	53	7870.9/100,000	5260/100,000
	40–44	13,000/100,000	22.30	24,400/100,000	54.08	7870.9/100,000	5260/100,000
	45–49	14,560/100,000	23.20	24,400/100,000	54.08	8107/100,000	5417.8/100,000
	50–54	16,307/100,000	24.13	24,400/100,000	55.16	8107/100,000	5417.8/100 000
	55–59	18,264/100,000	25.11	24,400/100,000	55.16	8350.2/100,000	5580.3/100,000
	60–64	20,446/100,000	26.13	24,400/100,000	56.92	8350.2/100,000	5580.9/100,000
	65–69	22,910/100,000	27.14	36,700/100,000	56.92	8600.7/100,000	5747.7/100,000
	70–74	25,659/100,000	28.19	36,700/100,000	58.05	8600.7/100,000	5747.7/100,000
	75–79	28,739/100,000	29.29	36,700/100,000	58.05	8600.7/100,000	5747.7/100,000

**Table 2:** Effectiveness of potassium and phytosterols to reduce the CVD RR.

	Age Group	RR Reduction Due to A 4.81% Decrease in LDL-c Levels [4,24]	RR Reduction Due to A 3.86 mmHg Decrease in SBP [66]	Compounded RR Reduction Due to Decreased LDL-c and SBP [4,24,66]	Compounded RR Ratio Due to Decreased LDL-c and SBP [4,24,66]
Stroke	35–44	1.96	20	21.96	78.04
	45–54	2.60	20	22.60	77.40
	55–64	2.61	16.5	19.11	80.89
	65–74	2.46	11.10	13.56	86.44
	75+	2.34	9	11.34	88.66
MI	35–44	1.96	16	17.96	82.04
	45–54	2.60	16	18.60	81.40
	55–64	2.61	12.5	15.11	84.89
	65–74	2.46	7.5	9.96	90.04
	75+	2.34	6	8.34	91.66

Data on the reduction in relative risk (RR) due to decreased LDL-c levels are a weighted average between hypercholesterolemic and normocholesterolemic subjects performed through age-stratified data on the prevalence of hypercholesterolemia in Malaysia. Given that no data were available on the severity of the hypercholesterolemic status of the different age cohorts, we assumed a uniform distribution between mildly and highly hypercholesterolemic subjects. Data on the decreased RR for stroke and MI due to increased potassium intake, originally available for 10 mmHg, were interpolated to a 3.68 mmHg reduction in SBP.

**Table 3:** Costs and utilities.

Outpatient Costs	Value (Range)
Yearly mean anti-hypercholesterolemic drug cost [90]	76.86 (21.86–133.86)
Yearly mean anti-hypertensive drug cost [90]	174.38 (19.38–329.38)
Screening visit cost, hypercholesterolemia [91]	24.03
Screening visit cost, hypertension [91,92,93,94,95]	7.38 (4.68–10.09)
Yearly number of screening visits, hypercholesterolemia [96]	1
Yearly number of screening visits, hypertension [97]	3
**Inpatient costs and days**	
Inpatient MI [82]	9491.00 (8395.18–10586.81)
Inpatient stroke [98]	4994.96 (2711.12–7278.33)
MI inpatient days, mean [7,99]	5.3
Stroke inpatient days, mean [98]	6.4
**Chronic costs**	
Chronic cost for the rest of year 1 MI [95,99,100]	305.23 (293.98–316.48)
Chronic cost after year 1 MI [95,99,100]	305.23
Chronic costs for the rest of year 1 stroke [101]	611.55
Chronic costs after year 1 stroke [101]	166.59
**Price of the milk powder product per portion/day**	0.5
**Discount (%)**	
Annual discount rate for costs and QALYs [89]	3
**Utilities (QALY)**	
MI [85]	0.45
Stroke [86]	0.56
Chronic CVD [87]	0.64
Well [88]	1
Death [88]	0

## Data Sharing

Requests for access to data should be made to the corresponding author (livia.dainelli@rdls.nestle.com).
